# Alvespimycin Inhibits Heat Shock Protein 90 and Overcomes Imatinib Resistance in Chronic Myeloid Leukemia Cell Lines

**DOI:** 10.3390/molecules28031210

**Published:** 2023-01-26

**Authors:** Raquel Alves, Diogo Santos, Joana Jorge, Ana Cristina Gonçalves, Steve Catarino, Henrique Girão, Joana Barbosa Melo, Ana Bela Sarmento-Ribeiro

**Affiliations:** 1 Laboratory of Oncobiology and Hematology (LOH), University Clinics of Hematology and Oncology, Group of Environmental Genetics of Oncobiology (CIMAGO), Faculty of Medicine (FMUC), University of Coimbra, 3000-548 Coimbra, Portugal; 2 Coimbra Institute for Clinical and Biomedical Research (iCBR), Faculty of Medicine (FMUC), University of Coimbra, 3000-548 Coimbra, Portugal; 3 Center for Innovative Biomedicine and Biotechnology (CIBB), 3004-531 Coimbra, Portugal; 4 Clinical Academic Center of Coimbra (CACC), 3000-370 Coimbra, Portugal; 5 Cytogenetics and Genomics Laboratory, Institute of Cellular and Molecular Biology, Faculty of Medicine (FMUC), 3000-548 Coimbra, Portugal; 6 Hematology Service, Centro Hospitalar Universitário de Coimbra (CHUC), 3004-561 Coimbra, Portugal

**Keywords:** heat shock protein, imatinib resistance, chronic myeloid leukemia

## Abstract

Heat shock protein 90 (HSP90) facilitates folding and stability and prevents the degradation of multiple client proteins. One of these HSP90 clients is BCR-ABL, the oncoprotein characteristic of chronic myeloid leukemia (CML) and the target of tyrosine kinase inhibitors, such as imatinib. Alvespimycin is an HSP90 inhibitor with better pharmacokinetic properties and fewer side effects than other similar drugs, but its role in overcoming imatinib resistance is not yet clarified. This work studied the therapeutic potential of alvespimycin in imatinib-sensitive (K562) and imatinib-resistant (K562-RC and K562-RD) CML cell lines. Metabolic activity was determined by the resazurin assay. Cell death, caspase activity, mitochondrial membrane potential, and cell cycle were evaluated by means of flow cytometry. Cell death was also analyzed by optical microscopy. HSPs expression levels were assessed by western blotting. Alvespimycin reduced metabolic activity in a time-, dose-, and cell line-dependent manner. Resistant cells were more sensitive to alvespimycin with an IC_50_ of 31 nM for K562-RC and 44 nM for K562-RD, compared to 50 nM for K562. This drug induced apoptosis via the mitochondrial pathway. In K562 cells, alvespimycin induced cell cycle arrest in G_0_/G_1_. As a marker of HSP90 inhibition, a significant increase in HSP70 expression was observed. Our results suggest that alvespimycin might be a new therapeutic approach to CML treatment, even in cases of resistance to imatinib.

## 1. Introduction

Chronic myeloid leukemia (CML) is a myeloproliferative neoplasia characterized by the presence of the *BCR–ABL1* fusion gene. The raised oncoprotein BCR–ABL presents constitutive tyrosine kinase activity that induces several downstream signaling pathways, as in the case of RAS/MAPK, PI3K/AKT, and JAK/STAT pathways, to promote cancer cell proliferation and survival [1]. The use of tyrosine kinase inhibitors (TKIs), such as imatinib, in CML treatment changed the clinical course of the disease dramatically [2]. Although the TKIs had clinical success, approximately 25% of CML patients developed drug resistance [3]. The molecular mechanisms of drug resistance acquisition can be multiple and are typically divided into mechanisms dependent on, or independent of, BCR–ABL [4]. To improve therapeutic approaches and clinical responses in TKI-resistant cases, it is necessary to identify and develop new therapeutic agents.

An important aspect of BCR–ABL oncogenic activity is its interaction with chaperone proteins, such as HSP90, to promote protein stability and prevent degradation [5]. Heat shock proteins (HSPs) are highly-conserved chaperones, expressed in response to stress conditions, and dysregulated in multiple neoplasias [6]. HSP90 is one of the most abundant proteins in eukaryotic cells, comprising 1–2% of cellular proteins under non-stress conditions. This chaperone is ATP-dependent and has an essential role in folding, activation, stabilization, and protection against proteasomal degradation of a wide range of proteins called client proteins [7]. HSP90 forms a multichaperone complex with HSP70, HSP40, P23, HOP (HSP organizing proteins), and ATP to perform these actions. In cancer cells, the expression of HSP90 is 2 to 10-fold higher than in normal cells, as described for CML cells [8]. 

Multiple oncoproteins have been identified as HSP90 client proteins, such as mutant c-KIT, HER2, mutant EGFR, BRAF, and BCR-ABL [8]. Targeting HSP90 to inhibit chaperone activity and promote client protein degradation may be an effective strategy in cancer treatment. A diversity of HSP90 inhibitors has been tested in CML and other neoplasias, including geldanamycin (GA) and its derivatives, tanespimycin (17 allylamino-17 demeth-oxygeldanamycin; 17-AAG) and alvespimycin (7 dimethylaminoethyla-mino-17-demethoxygeldanamycin; 17-DMAG) [5]. These drugs competitively bind to the ATP binding site of HSP90, leading to multichaperone complex dissociation and, consequently, client protein degradation [9]. Alvespimycin is a semi-synthetic derivative of GA with higher water solubility, better bioavailability for oral use, a longer plasma half-life, and less extensive metabolism [10]. Due to these improved characteristics, this inhibitor has been tested in different neoplasias with relevant results. However, the role of this drug in cancer drug resistance, namely in CML resistant to imatinib, is not yet clarified.

In this context, we investigated the effect of alvespimycin as a targeted therapy in imatinib-sensitive and imatinib-resistant CML cell lines. This inhibitor induced cell death of CML cells through apoptosis activation with a higher effect in resistant models. 

## 2. Results

### 2.1. Alvespimycin Decreases the Metabolic Activity of CML Cell Models

**Figure 1:**
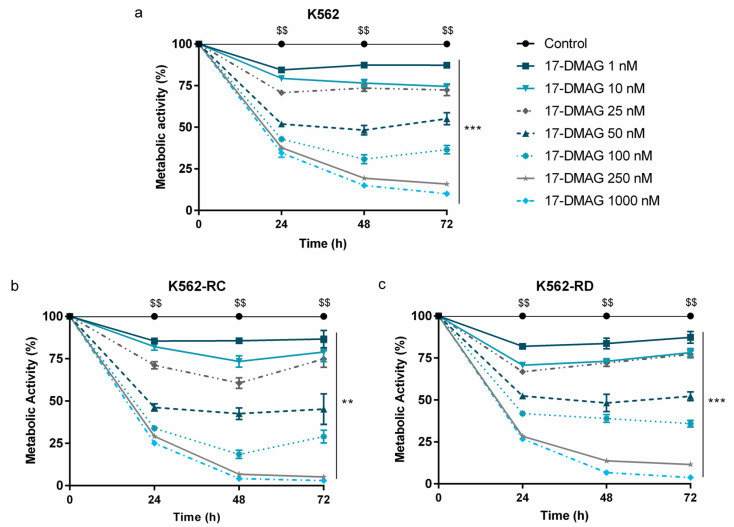
Dose–response curves of alvespimycin on imatinib-sensitive and imatinib-resistant CML cell lines. The sensitive cell line K562 (**a**), and the imatinib-resistant cells K562-RC (**b**), and K562-RD (**c**) cells were incubated in the absence of, and in presence of, different concentrations of alvespimycin in monotherapy for 72 h. Results were expressed in percentage (%) normalized to control and represent the mean ± SEM obtained from 6 independent experiments. After 48 h of exposure to alvespimycin, K562 cells presented a mathematical IC_50_ of 50 nM, the K562-RC cells of 31 nM, and K562-RD cells of 44 nM. Control corresponds to cells with drug solvent (DMSO). ** *p* < 0.01; *** *p* < 0.001 (comparison with control); $$ *p* < 0.01 (comparison with time 0 h).

The HSP90 inhibitor, alvespimycin, decreased the metabolic activity in a time-, dose- and cell line-dependent manner, as represented in Figure 1. The imatinib-sensitive K562 cells presented a mathematical IC_50_ of 50 nM at 48 h (Figure 1a). A more pronounced effect was observed in imatinib-resistant CML models (Figure 1b,c). After the same exposure time, the IC_50_ of K562-RD was 44 nM and of K562-RC was 31 nM; this last model being the most sensitive to alvespimycin. Especially in resistant models, after 48h of incubation, a recovery in the metabolic activity of the cells incubated with the drug was observed in some concentrations. Despite this, in all models, alvespimycin in the concentration of 250 nM, or higher, decreased the metabolic activity of cells to 25% after 48 h of incubation.

**Figure 2:**
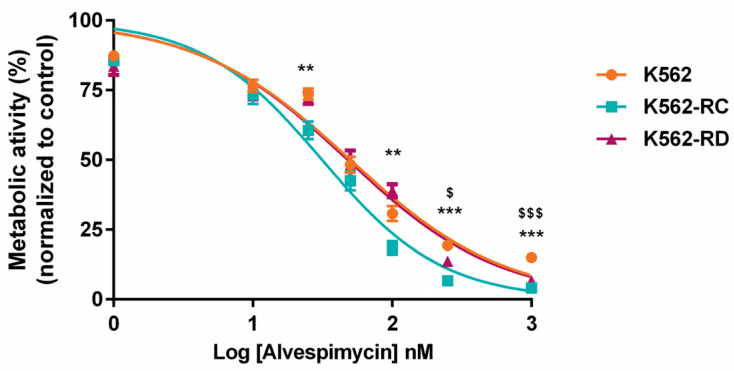
Effect of alvespimycin in metabolic activity of K562, K562-RC, and K562-RD cells. Cells were incubated in the absence of, and in the presence of, different concentrations of alvespimycin for 48 h. The curves represent the behavior of each cell line to increasing doses of HSP90 inhibitor. The results were expressed in percentage (%) normalized to control (cells treated with drug solvent) and represent the mean ± SEM obtained from 6 independent experiments. ** *p* < 0.01, *** *p* < 0.001 (comparison of K562-RC with K562); $ *p* < 0.05, $$$ *p* < 0.001 (comparison of K562-RD with K562).

Comparing the effect of alvespimycin across the different cell lines (Figure 2), we observed a statistically significant difference between K562-RC cells and K562 for doses superior to 100 nM. In the same line, a more pronounced effect was observed in K562-RD for doses superior to 250 nM of alvespimycin, compared with the imatinib-sensitive cell line.

### 2.2. Apoptosis as the Cell Death Mechanism Triggered by Alvespimycin 

**Figure 3:**
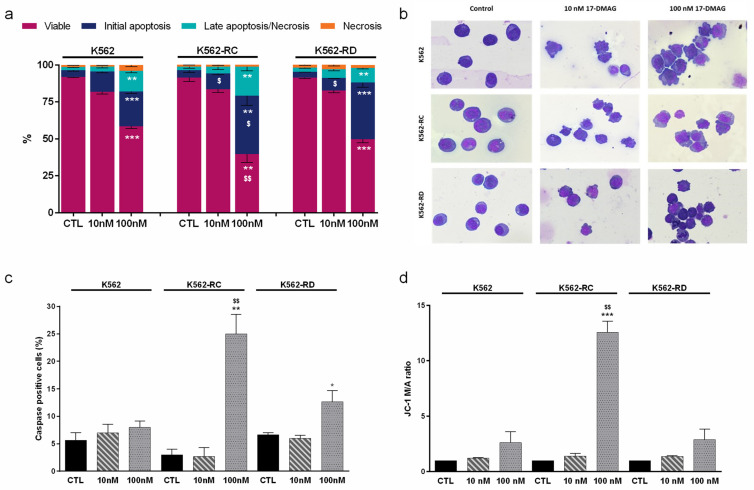
Analysis of cell death induced by alvespimycin in imatinib-sensitive (K562) and imatinib-resistant (K562-RC and K562-RD) CML cell lines. (**a**) The type of cell death was identified by annexin V/7-AAD staining and analyzed by flow cytometry; data were expressed as a percentage (%) of live, early apoptotic, late apoptotic/necrosis, and necrosis. In (**b**), cell morphology was analyzed in smears stained with May–Grünwald–Giemsa (amplification: 500×). In (**c**), the caspase expression levels were analyzed using the Apostat probe, and in (**d**), the ΔΨ_mit_ was analyzed using JC-1 fluorescent probe. JC-1 probe coexists in monomeric (M) or aggregate (A) forms depending on the mitochondrial membrane potential. The ratio between M/A JC-1 forms expresses ΔΨmit results. The results were obtained after 48 h of incubation and represent mean ± SEM of three independent experiments. Control (CTL) corresponds to cells with drug solvent (DMSO). * *p* < 0.05, ** *p* < 0.01, *** *p* < 0.001 (comparison with respective cell line control); $ *p* < 0.05, $$ *p* < 0.01 comparing with the same condition of sensitive K562 cells.

We evaluated the mechanism of cell death induced after 48 h of incubation with alvespimycin in the concentration of 10 nM and 100 nM (Figure 3). Using Annexin V/PI staining (Figure 3a), we observed that this drug induced a dose-dependent decrease in viable cells, along with an increase in apoptotic cells. The increase in early apoptosis and late apoptosis/necrosis populations confirmed the apoptosis activation. For the highest dose of alvespimycin, the differences in the decrease of the population of viable cells were statistically significant in all cell lines, compared with respective control cells (*p* < 0.01 for K562-RC; *p* < 0.001 for K562 and K562-RD). Moreover, the apoptosis activation was more pronounced in the resistant models (K562-RC: 39.4 ± 6.4% and K562-RD: 38.4 ± 3.2%), compared to the sensitive model (K562: 23.6 ± 1.3%), after exposure to 100 nM of alvespimycin. Comparing the effect of 100 nM across all cell lines, we observed a significant reduction in the viable population of K562-RC cells, compared to K562 ($ *p* < 0.01). In agreement with the AV/PI assay results, the morphological analysis showed morphological features typical of apoptosis, such as cellular contraction, nuclear fragmentation, and blebbing in cells exposed to alvespimycin (Figure 3b).

For further confirmation of apoptosis induction, we assessed the caspases expression levels (Figure 3c) and analyzed the mitochondrial membrane potential (Figure 3d). We observed an increase in caspases positive cells in the presence of the HSP90 inhibitor, with a superior effect in the highest concentrations. The K562-RC cells presented the highest levels, with 25% of caspase-positive cells (*p* < 0.01), followed by K562-RD (≈13%; *p* < 0.05) and K562 cells (≈8%) for cells treated with 100 nM of alvespimycin when compared to respective control cells. Furthermore, for 100 nM of alvespimycin condition, K562-RC presented a significant increase in caspase-positive cells compared to imatinib-sensitive cells ($$ *p* < 0.01).

Since mitochondria can be involved in apoptosis, we analyzed the mitochondrial membrane potential using JC-1 dye (Figure 3d). Apoptotic cells exhibit a higher JC-1 monomer/aggregate ratio (M/A), corresponding to a decrease in mitochondrial membrane potential, than viable cells. Alvespimycin induced a dose-dependent increase in the M/A JC-1 ratio in all cell lines. The decrease of mitochondrial membrane potential was only statistically significant for the K562-RC exposed to 100 nM of alvespimycin, with an approximately 12 times higher M/A ratio compared to the respective control condition (*p* < 0.001) and to equally treated sensitive cell line ($$ *p* < 0.01).

### 2.3. Alvespimycin Promotes Cell Cycle Arrest in G_0_/G_1_ Phase

In order to study if alvespimycin has a cytostatic effect in addition to its cytotoxic potential, we analyzed the cell cycle distribution (Table 1). In the sensitive cell line (K562), we observed a significant cell cycle arrest in the G_0_/G_1_ phase in cells treated with 100 nM of this drug (40.0% at control vs. 60.3% in alvespimycin 100 nM treated cells; *p* < 0.01). A similar pattern was observed in K562-RC cells, with an increase in the % of cells in the G_0_/G_1_ phase. However, the differences did not reach significant levels. In resistant K562-RD cells, no significant differences were denoted, compared to the control, but a slight increase in the G_2_/M phase was detected.

By the same analysis, it was possible to identify a sub-G_1_ peak that corresponded to DNA fragmentation, typical of apoptotic cells. The HSP90 inhibitor promoted a dose-dependent increase in the sub-G_1_ population in all cell lines, supporting our previous results. For cells treated with 100 nM of alvespimycin condition, the differences were statistically significant, with higher levels in K562-RC cells (17.4%; *p* < 0.01)

### 2.4. Heat Shock Proteins Expression

**Figure 4:**
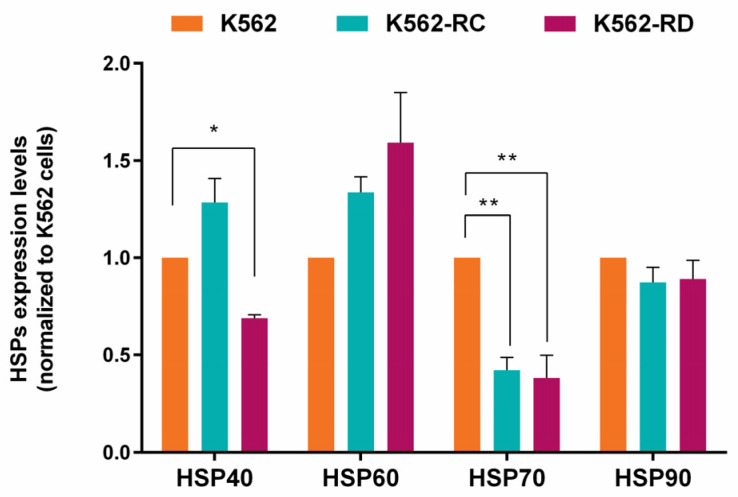
Heat shock proteins (HSP) baseline expression in imatinib-sensitive and imatinib-resistant CML cell lines. The densitometric analysis was performed using ImageJ software, and the values obtained represent the ratio between the immunodetected protein and the loading control (α-Tubulin). The results are normalized to imatinib-sensitive cells (K562) and are expressed as the mean ± SEM of 3 independent experiments. * *p* < 0.05, ** *p* < 0.01 comparing with K562.

Since the study focused on HSP90 inhibition, we assessed the baseline expression levels of different HSP proteins in imatinib-sensitive and imatinib-resistant cells (Figure 4). Regarding HSP90 expression, resistant cells presented a slightly lower expression than K562 cells but without statistical significance. A significant difference was found in HSP70-1 expression levels with a reduction in imatinib-resistant cells, compared to sensitive K562 cells (*p* < 0.01). Even without statistical meaning, the resistant models presented the highest levels of HSP60. In HSP40 expression, an opposite pattern was observed between the resistant cell lines, with the K562-RC cells presenting the highest levels and the K562-RD having the lowest expression (*p* < 0.05 compared to K562).

**Figure 5:**
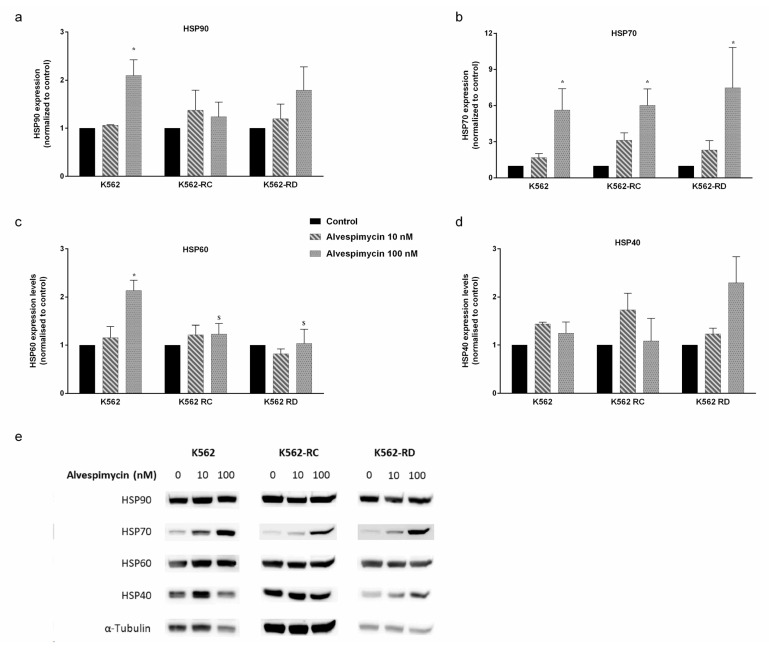
HSP expression in CML cell lines after alvespimycin exposure. The effect of alvespimycin on HSP90 (**a**), HSP70-1 (**b**), HSP60 (**c**), and HSP40 (**d**) after 48 h of alvespimycin exposure. In (**e**) representative western blots analysis of HSPs. The densitometric analysis was performed using ImageJ software, and the values obtained represent the ratio between the immunodetected protein and the loading control (α-Tubulin). The results are expressed as the mean ± SEM of 3 independent experiments. Control corresponds to cells with drug solvent (DMSO). * *p* < 0.05 compared to the respective cell line control; $ *p* < 0.05 comparing with the same condition of sensitive K562 cells.

The same protein expression analysis was performed after 48 h of incubation with alvespimycin (Figure 5). In general, this drug induced a dose-dependent increase in HSPs expression in all cell lines. In imatinib-sensitive cells, the treatment with 100 nM of alvespimycin induced a 2×-fold expression of HSP90 (*p* < 0.05; Figure 5a), 5×-fold expression of HSP70 (*p* < 0.05; Figure 5b), 2×-fold expression of HSP60 (*p* < 0.05; Figure 5c), and a slight increase in HSP40 expression (Figure 5d), compared to control cells. Similar behavior was denoted in resistant cells, where the differences in HSP70-1 were more pronounced with a significant increase of 5×- and 7.5×-fold more expression in K562-RC (*p* < 0.05) and K562-RD (*p* < 0.05), respectively, compared with untreated cells. Additionally, we observed differences in the effect of 100 nM of alvespimycin across the three cell lines for the HSP60 expression, being lower in imatinib-resistant cells compared to K562 ($ *p* < 0.05).

## 3. Discussion

The activity of HSP90 and other HSP proteins is crucial for the activity of oncoproteins like BCR–ABL. Based on this, these proteins became an attractive target for cancer. In this study, we evaluated the therapeutic potential of an HSP90 inhibitor, alvespimycin, in monotherapy in CML in in vitro models sensitive and resistant to imatinib. This study demonstrated that resistant models were more sensitive to alvespimycin than the parental sensitive cells (K562), with the effect being time- and dose-dependent in manner. In our models, we saw a reversion in the effect of alvespimycin for some concentrations, indicating the need to adjust the drug administration scheme to achieve more pronounced results. Regarding the mechanism of action, alvespimycin activated apoptosis through the mitochondrial pathway. This drug also induced a cell cycle arrest in the G0/G1 phase in sensitive cells. The inhibition of HSP90 led to a heat shock response with an increase of HSPs proteins after drug exposure.

HSPs significantly impact tumorigenesis by interfering with several signaling pathways that culminate in cancer cell proliferation, survival, and metastization [11]. Over the years, multiple HSP inhibitors have been developed for the different HSPs, several designed to target HSP90 [8,11]. The potential of these drugs relies on higher affinity to cancer cells compared to normal cells. For 17-AAG, a derivative of GA as alvespimycin, Kamal et al. described a 100-fold higher binding affinity in cancer cells than in healthy cells [12]. In this study, we selected alvespimycin as an HSP90 inhibitor to test, considering its better pharmacokinetic features that make this drug more promising for clinical applications [5]. One relevant characteristic of alvespimycin was the lower toxicity observed in normal hematopoietic cells, described by Hertlein et al. [13]. These authors, for concentrations of alvespimycin from 100 nM to 1 μM, reported no cytotoxicity in T and NK cells and minimal toxicity in normal B cells [13]. In the same line, Ikebe et al. evaluated the alvespimycin effect in normal peripheral blood leukocytes (from 0.1 μM to 10 μM) and did not observe a drug effect in these cells [14]. Particularly in CML, HSP90 expression levels were described as higher in blast crisis patients, and according to Zacková et al., this can be a prognostic marker of response [15]. Low levels of HSP90 were associated with good response cases, while high levels were found in resistant patients [15]. In our models, we did not detect significant differences in HSP90 expression levels between sensitive and resistant models, other than being slightly lower in resistant cells. These results could be related to the molecular mechanism that mediated imatinib resistance acquisition. As previously described, the K562-RC and K562-RD models mainly presented alterations in drug transporters and alternative signaling pathways independent of BCR–ABL [16,17]. Consistently, other authors have suggested that, in vitro, the stability of BCR–ABL was more dependent on HSP90 in the case of imatinib-resistant mutations [18]. Even so, our imatinib-resistant cells were more sensitive to alvespimycin, possibly due to the ability of HSP90 inhibition to simultaneously affect multiple oncogenic pathways essential for the resistant phenotype. Similar sensitivity to alvespimycin was also described in gemcitabine-resistant and 5-fluorouracil-resistant pancreatic cancer and lapatinib-resistant breast cancer [19,20]. Furthermore, alvespimycin has been evaluated in other hematological malignancies, such as diffuse large B-cell lymphoma (DLBCL), chronic lymphocytic leukemia, and acute myeloid leukemia (AML). The drug was assessed in a clinical trial setting in these last two pathologies [21,22,23]. Additionally, over the last years, different HSP90 inhibitors have been tested in imatinib-resistant models, such as 17-AAG, BIIB021, and KW-2478, with similar results to those we obtained, although some of the drugs required higher doses [24,25,26].

According to the literature, apoptosis was the cell death mechanism activated by alvespimycin associated with increased caspases and decreased mitochondrial membrane potential. Rao et al. demonstrated that alvespimycin treatment inhibited activated TRKA and its downstream signaling through p-AKT and p-ERK1/2, resulting in apoptosis of cell lines and primary human AML and CML cells [27]. In the same line, in DLBCL cell lines, this inhibitor induced apoptosis with a decreased mitochondrial membrane potential [21]. Other GA derivatives and other HSP90 inhibitors share this mechanism of action through apoptosis induction [24,25,26,28,29]. For instance, in mantle cell lymphoma cell lines, 17-AAG treatment was associated with the activation of the intrinsic apoptosis pathway [30]. Some authors point out that the proteasomal degradation of PI3K/AKT, NF-kB, and RAS/MAPK signaling pathway members was the apoptotic trigger of GA derivatives [31].

Complementary to the cytotoxic effect, alvespimycin also interferes with cell cycle distribution, particularly in K562 cells. As described in gastric cancer cells [32], alvespimycin induces significant G0/G1 arrest in imatinib-sensitive cells and a similar tendency in K562-RC. This effect could be justified by the influence of HSP90 action on stabilization cyclin D and CDK4 that is abrogated in treated cells [30,33,34]. For other HSP90 inhibitors, an accumulation of cells in G_0_/G_1_ was also described, accompanied by an increase in p21 expression levels [35]. In the grade IV bladder cancer cell line, TCCSUP, the exposure to alvespimycin induced a cell cycle arrest in G2/M that could result from dysregulation of HSP90 client proteins involved in this cell cycle checkpoint [34]. A similar tendency was observed in our K562-RD cells. 

The function of HSP90 relies upon the formation of a multichaperone complex with other HSPs, as in the case of HSP70 and HSP40 [10]. Considering this, we evaluated the expression levels of four HSPs in baseline conditions and after alvespimycin treatment. As previously discussed for HSP90, we found that HSP70 was downregulated in resistant models. In the literature, the expression of this chaperone in imatinib-resistant models is not consensual. As described by us, Lee et al. reported that the antiapoptotic proteins HSP70 and STAT5 were significantly downregulated in imatinib-resistant variants [36]. On the contrary, other authors demonstrated a 3x-fold increase in HSP70 expression levels in imatinib-resistant cells versus the sensitive cell line [37]. This variability may be related to different mechanisms of resistance activated in each imatinib-resistant model. Additionally, multiple authors reported the expression of HSP70 and HSP40 as functional markers of HSP90 inhibition [19,29,38]. The inhibition of HSP90 could lead to a heat shock response that, by a negative feedback loop, leads to activation of the heat shock transcription factor (HSF1), which causes transcriptional induction of HSP70, HSP40, HSP27, and, to a smaller degree, of HSP90 itself, trying to protect cancer cells from apoptosis [38]. In accordance with the higher doses of alvespimycin, we observed significant alterations in HSP proteins, especially in HSP70 expression levels. Moreover, in K562 cells exposed to 100nM of alvespimycin, we observed an increase in HSP60 expression, known by its relevant role in mitochondria, that could result from a compensatory mechanism to reestablish mitochondrial function impaired by the drug [39].

The resistance to targeted therapies is an emerging problem in cancer management that can be overcome with new drugs and combinatorial drug schemes. Preclinical evidence indicates that silencing the cochaperones HSP70, HSP27, or HSF-1 is associated with increased sensitivity to HSP90 inhibition [40]. Additionally, HSP90 inhibitors have shown either additive or synergistic activity in combination with various chemotherapeutic agents, including gemcitabine, carboplatin, docetaxel, and irinotecan, and with radiotherapy [9,26,28,40]. These promising results on CML-resistant models support further investigation of the potential of alvespimycin to resensitize CML cells to TKIs. Moreover, this opens the possibility of testing new combination schemes with other agents using lower doses that could synergistically induce CML cell death. 

## 4. Materials and Methods

### 4.1. Cell Lines and Cell Culture Conditions

This study used the K562 cell line as a CML model sensitive to imatinib and two imatinib-resistant CML cell lines—K562-RC and K562-RD cells. K562 cells were purchased from American Type Culture Collection (ATCC), and the imatinib-resistant cells were developed in our laboratory, based on continuous (K562-RC) and discontinuous exposure (K562-RD) to imatinib (Selleck Chemicals, Houston, TX, USAm), as described in Alves et al. [16]. The cell lines were maintained in RPMI-1640 medium supplemented with 10% FBS, 2 mM of L-glutamine, 100 U/mL of penicillin, and 100 μg/mL of streptomycin (Gibco, Invitrogen, Waltham, MA, USA) at 37 °C in a humidified atmosphere containing 5% of CO_2_. Based on different resistance schemes, 250 nM of imatinib was added to the medium of resistant cell lines. For sensitive cells, the IC_50_ of imatinib was 75 nM, while the IC_50_ of K562-RC was 605 nM, and 1390 nM for K56-RD cells.

### 4.2. Metabolic Activity Assay

The metabolic activity of cells was determined using the resazurin assay in the absence of, and in the presence of, increasing concentrations (from 1 nM to 1000 nM) of alvespimycin (Selleck Chemicals, Houston, TX, USA) in a single administration. The inhibitor was diluted in dimethyl sulfoxide (DMSO), and the same amount of solvent was added to each condition tested. The control (CTL) conditions corresponded to cells treated with DMSO. Briefly, the cells were plated at 0.5 × 10^6^ cells/mL, and after the treatment, resazurin was added to a final concentration of 10 μg/mL to the cells for 2 h incubation. The absorbance was measured at 570 nm and 600 nm, and the metabolic activity was calculated as a percentage of control. The results were expressed as a mean ± standard error of the mean (SEM) of six independent experiments.

### 4.3. Assessment of Cell Death

Cell death was determined by optical microscopy, using the May–Grunwald–Giemsa staining, and by flow cytometry (FC), using the Annexin-V (AV) and propidium iodide (PI) double staining. For both analyses, the cells were treated with 10 nM and 100 nM of alvespimycin and incubated for 48 h. After that, the cells were washed with PBS by centrifugation at 500× *g* for 5 min. In flow cytometry assays, the cells were resuspended in 100 µL of binding buffer and incubated with 5 µL of AV-APC (Biolegend, San Diego, CA, USA) and 2.5 µL of PI (Immunostep, Salamanca, Spain) for 15 min in the dark. Then, cells were diluted in 400 µL of binding buffer and analyzed by FC. The analysis was performed in a FACSCalibur™ (Becton Dickinson, Franklin Lakes, NJ, USA), where at least 25.000 events were acquired using CellQuest software (Becton Dickinson, Franklin Lakes, NJ, USA) and analyzed using Paint-a-Gate (Becton Dickinson, Franklin Lakes, NJ, USA). The results corresponded to the percentage of each cell population: viable cells (AV−/PI−), early apoptotic (AV+/PI−), late apoptotic/necrotic (AV+/PI+), and necrotic cells (AV−/PI+), and represented the mean ± SEM of five independents experiments. For optical microscopy assays, the cell smears were stained with May–Grunwald–Giemsa solution (Sigma-Aldrich, St. Louis, MO, USA), as described by Mendes et al. [41]. Cell morphology was evaluated by light microscopy using a Nikon Eclipse 80i equipped with a Nikon Digital Camera DXm 1200F (Nikon, Tokyo, Japan).

### 4.4. Evaluation of Caspases Activity

The Apostat probe (R&D Systems, Minneapolis, MN, USA) was designed to identify and quantify cell caspase activity by FC. Cells were incubated for 48h in the absence of (cells with DMSO), or the presence of, 10 nM and 100 nM of alvespimycin. For each condition, 1 × 10^6^ cells were resuspended in 1000 µL of PBS and incubated with 1 µg of Apostat. After a 15 min incubation period at 37 °C, the cells were washed and resuspended in 400 µL of PBS for FC analysis. The results are expressed in percentage (%) and represented as mean ± SEM of three independent experiments. 

### 4.5. Cell Cycle Analysis

Cell cycle evaluation was performed in cells in the absence of (cells with DMSO), or in the presence of, 10 nM and 100 nM of alvespimycin after 48 h of incubation, utilizing PI solution with RNAse, according to manufacturer instructions. For each condition, 1 × 10^6^ cells were collected and washed with PBS for 5 min at 1000 g. The pellet was resuspended in 200 µL of 70% ethanol solution during vortex agitation and incubated for 30 min at 4 °C. Then, cells were washed with PBS, resuspended in 500 µL of PI/RNase solution (Immunostep, Salamanca, Spain), and finally analyzed by FC. The results were expressed as a percentage of cells in each cycle phase (G_0_/G_1_, S, G_2_/M) after the analysis by ModFit LT software (Verity Software House, Topsham, ME, USA). In addition, the sub-G_1_ population was also identified and corresponded to apoptotic cells. The results represent the mean ± SEM of five independent experiments.

### 4.6. Mitochondrial Membrane Potential Assessment

Mitochondrial membrane potential (ΔΨ_mit_) was evaluated using the fluorescent probe 5,5′,6,6′-tethrachloro-1,1′3,3′-tetraethylbenzimidazolcarbocyanine iodide (JC-1; Molecular probes, Eugene, OR, USA). JC- 1 is a lipophilic cationic probe that exists in a monomeric form (M) emitting at 527 nm (green fluorescence), and it can reversibly form aggregates (A), which emit 590 nm (greenish-orange fluorescence) as the mitochondrial membrane becomes more polarized. In apoptotic cells, mitochondrial membrane potential collapses, and JC-1 cannot accumulate within the mitochondria, remaining in the monomeric form in cytosol. These cells exhibit a higher monomer/aggregate ratio of JC-1 (M/A) than viable cells [42]. To determine the ΔΨ_mit_, the cells were treated with 10 nM and 100 nM of alvespimycin and incubated for 48 h. For each condition, 1 × 10^6^ cells were collected, washed, resuspended in 1000 µL of PBS, and incubated with JC-1 (5 μg/mL) for 15 min at 37 °C in the dark. At the end of the incubation period, the cells were washed twice in PBS, resuspended in 300 μL, and analyzed by FC. The results were expressed in mean ± SEM of monomer/aggregate ratio of JC-1 of three independent experiments. The M/A ratio was calculated as the mean intensity fluorescence (MIF) fraction observed for each JC-1 form.

### 4.7. Heat Shock Protein Expression Analysis

HSP expression analysis was performed on cells in the absence of (cells with DMSO), or in the presence of, 10 nM and 100 nM of alvespimycin after 48 h of incubation, using HSP/Chaperone antibody kit (Cell Signaling Technology, Danvers, MA, USA). In more detail, we used HSP40 (CST #4871), HSP60 (CST #12165), HSP70-1 (CST #4872), and HSP90 (CST #4877) antibodies to evaluate total HSP expression levels. After incubation, the cells were washed with PBS and resuspended in RIPA buffer (10 mM Tris base, 0.25 M saccharose, and 1 mM EDTA in the presence of protease inhibitors). The lysates were sonicated and then centrifuged at 4 °C, 10,000 g for 10 min. The supernatants were analyzed for protein content using a bicinchoninic acid assay (BCA) kit (Thermo Fisher Scientific, Waltham, MA, USA). For the immunodetection of proteins, 30 μg of total cell protein was separated by electrophoresis on 10% SDS polyacrylamide gels and transferred to nitrocellulose membranes. Next, the membranes were blocked with 5% non-fat milk in Tris-buffered saline with 0.1% Tween-20 (TBS-T) for 1 h and incubated with specific primary antibodies overnight at 4 °C. At the end of this incubation, membranes were washed with TBS-T and incubated with peroxidase-conjugated antibodies. The HSP40, HSP60, HSP70-1, HSP90 (1:1000), and α-Tubulin (1:10,000, Sigma-Aldrich, St. Louis, MO, USA) were detected using ECL and ImageQuant imaging system (GE Healthcare, Chicago, IL, USA). The densitometric analyses were performed using ImageJ software, and the values obtained represented the ratio between the immunodetected protein and loading control (α-Tubulin) of three independent experiments.

### 4.8. Statistical Analysis

Statistical analysis was performed using GraphPad Prism version 7.00 (GraphPad Software, San Diego, CA, USA). Data are expressed in mean ± SEM of indicated independent experiments. The IC_50_ determination was performed by non-linear curve fit dose-response. The Kolmogorov–Smirnov test was used to assess normality, and adequate analysis was used in accordance. Kruskal–Wallis test and one-way ANOVA were used to determine the statistical significance, followed by Dunnett’s and Dunn’s as multiple comparison tests. A significance level of *p* < 0.05 was considered statistically significant.

## 5. Conclusions

Our results highlight the role of HSP90 as a valuable target in CML and support the potential application of alvespimycin as a new therapeutic approach for CML, especially in cases of resistance to imatinib.

## Figures and Tables

**Table 1:** Effects of alvespimycin in the cell cycle of sensitive and imatinib-resistant cells.

	sub-G_1_ (%)	G_0_/G_1_ (%)	S (%)	G_2_/M (%)
***K562 Cells***				
Control	1.4 ± 0.2	40.0 ± 2.4	44.2 ± 3.9	15.8 ± 1.8
Alvespimycin 10 nM	5.6 ± 2.9	40.1 ± 0.7	46.4 ± 1.2	13.5 ± 0.9
Alvespimycin 100 nM	9.8 ± 2.0 **	60.3 ± 5.4 **	21.3 ± 7.3	18.4 ± 2.0
***K562-RC Cells***				
Control	0.2 ± 0.2	52.4 ± 3.6	33.6 ± 3.0	14.0 ± 0.8
Alvespimycin 10 nM	0.6 ± 0.4 ^$^	52.6 ± 2.5	33.6 ± 2.6	13.8 ± 0.2
Alvespimycin 100 nM	17.4 ± 5.2 **	62.0 ± 5.8	28.5 ± 3.2	9.5 ± 3.4
***K562-RD Cells***				
Control	0.2 ± 0.2	44.4 ± 1.5	36.6 ± 2.6	19.0 ± 2.2
Alvespimycin 10 nM	0.8 ± 0.4 ^$^	45.0 ± 2.2	36.4 ± 2.5	18.6 ± 1.7
Alvespimycin 100 nM	3.0 ± 0.8 **	41.3 ± 1.4	32.7 ± 3.8	26.0 ± 4.7

Control corresponds to cells with drug solvent (DMSO). ** *p* < 0.01 comparing with respective cell line control; ^$^ *p* < 0.05 comparing with the same condition of sensitive K562 cells.

## Data Availability

All data generated or analyzed during this study were included in this published article.
